# Application of the health belief model to study weight management behavioral intentions among adults in Ho Chi Minh City, Vietnam: a cross-sectional study

**DOI:** 10.1186/s12889-025-23637-9

**Published:** 2025-07-11

**Authors:** Thang T. Vo, Quoc-Duy Le

**Affiliations:** 1 Health and Agricultural Policy Research Institute (HAPRI), University of Economics Ho Chi Minh City, Floor 10, 279 Nguyen Tri Phuong, District 10, Ho Chi Minh City, Vietnam; 2 School of Economics, University of Economics Ho Chi Minh City, Ho Chi Minh City, Vietnam

**Keywords:** Health belief model, Weight management behavioral intention, Overweight, Obesity, Vietnam

## Abstract

**Background:**

The prevalence of overweight and obesity is rapidly increasing globally, particularly in developing countries such as Vietnam, leading to heightened risks of chronic diseases and significant economic burdens. Although various weight management strategies have been implemented, success rates remain low due to the lack of sustained adherence.

**Objective:**

This study aims to identify factors influencing the behavioral intentions for weight management among adults in Ho Chi Minh City through the Health Belief Model (HBM) and assess their impact levels.

**Methods:**

A cross-sectional study was conducted on 387 adults recruited in Ho Chi Minh City from July to October 2023, using a stratified quota sampling technique based on age and educational attainment. Data were collected through an online questionnaire measuring the components of the HBM and the basic characteristics of the participants. Multivariable logistic regression analysis was performed to determine the factors influencing behavioral intentions for weight management.

**Results:**

Perceived threat of overweight (β = 0.92, OR = 2.51), perceived self-efficacy in exercise (β = 0.87, OR = 2.39), female sex (β = 1.14, OR = 3.13), educational attainment at secondary school (β = 1.32, OR = 3.73), high school (β = 1.56, OR = 4.73), college/university graduate or higher (β = 1.98, OR = 7.25), dieting experience (β = 0.61, OR = 1.84), and BMI (β = 0.26, OR = 1.29) were significantly positively associated with the intention to manage weight (*p* < 0.05). In contrast, marital status (being married) was the only factor negatively associated with weight management intentions (β = -1.12, OR = 0.33, *p* = 0.002).

**Conclusion:**

The study identified the perception of overweight threat, self-efficacy in exercise, female sex, higher educational attainment, weight loss experience through dieting, and BMI as key factors driving weight management behavioral intentions, whereas marital status (being married) had a negative impact. These findings suggest that weight management interventions in Vietnam should focus on enhancing awareness of overweight risks, improving self-efficacy in exercise, and personalizing intervention strategies to optimize effectiveness.

**Supplementary Information:**

The online version contains supplementary material available at 10.1186/s12889-025-23637-9.

## Introduction

Overweight and obesity have become significant global health burdens [1–4], contributing to the onset of numerous chronic diseases such as type 2 diabetes [5, 6], cardiovascular diseases [5, 7], osteoarthritis [8, 9], and certain cancers [10–13]. This condition is defined by abnormal or excessive accumulation of body fat, with common diagnostic criteria including body mass index (BMI ≥ 25 for overweight, ≥ 30 for obesity), waist circumference, waist-to-hip ratio, and body fat percentage [14]. Excess fat accumulation not only increases body weight but also leads to insulin resistance, chronic systemic inflammation, dyslipidemia, and endocrine disturbances, key pathophysiological mechanisms that elevate the risk of metabolic and cardiovascular diseases [15, 16].

According to the World Health Organization (WHO), approximately 2.5 billion adults aged 18 and over were overweight in 2022, with at least 850 million categorized as obese [14]. The COVID-19 pandemic has exacerbated this issue, as lockdowns and restrictions have led to reduced physical activity and changes in eating habits, increasing the risk of overweight and obesity [17, 18]. Without effective prevention and treatment measures, according to the current trend, more than half of the global population could be overweight or obese by 2035, with economic impacts potentially reducing global GDP by 2.9% [19].

The significant rise in overweight and obesity is not limited to developed countries but has also become a major challenge for developing nations, including Vietnam [20, 21]. With a population of approximately 98 million people, Vietnam has made remarkable economic strides, with a per capita GDP of nearly 4,347 USD in 2023 [22], and a sharp reduction in the poverty rate from over 14% in 2010 to 4.2% in 2020 [23]. Alongside economic development, the diet of the population has improved [24, 25], leading to significant changes in Body Mass Index (BMI) [26]. A meta-analysis from 1998 to 2020 revealed that the prevalence of overweight among adults in Vietnam was 20.3% [27]. Despite being lower than the global average (43%) [14], this rate has grown significantly, increasing from 6.6% in 2005 to 20.3% in 2020 [27, 28]. Furthermore, the 2015 STEPS survey indicated that nearly one-third of the Vietnamese population did not meet the WHO-recommended levels of physical activity, and 15.6% were overweight or obese, with a rising trend [29]. The prevalence of overweight and obesity in Vietnam is rapidly increasing [30], with a significantly higher rate in urban adults (21.3%) compared to rural areas (12.6%) [29]. According to the World Obesity Federation, the annual increase in obesity among adults (> 19 years) is projected at 6.3%, and among children (< 5 years) at 9.8%, reaching alarming levels by 2035 [19].

Obesity results from the complex interaction between genetic predisposition, individual behaviors, and environmental factors, with unhealthy diets and physical inactivity being the primary contributors [31]. Although pharmacological and surgical treatments may be effective in specific cases, lifestyle interventions, particularly those involving behavioral changes in conjunction with diet and physical activity, remain the most sustainable and widely adopted approaches to weight control among adults [32, 33]. However, achieving successful weight loss remains a significant challenge, with low rates of long-term adherence and maintenance [34–36]. Previous studies have highlighted that psychosocial factors, such as health and obesity perceptions and self-efficacy, play critical roles in the success of weight loss and maintenance programs [37–39]. Therefore, to develop effective weight management interventions for adults, it is crucial to identify the factors influencing weight management behavioral intentions.

The Health Belief Model (HBM) has been widely applied to explain weight management behavior, based on perceptions reflecting an individual’s readiness to adopt health-protective behaviors [40–42]. Specifically, HBM includes factors such as perceived susceptibility, severity, barriers, benefits, cues to action, and self-efficacy, which help predict and explain why individuals change or maintain specific health behaviors [43, 44]. Notably, the combination of perceived susceptibility and severity forms the construct of perceived threat [41, 42]. Numerous studies have examined factors influencing weight management intentions through the HBM [41, 42, 45, 46]. Research has shown that perceived threat [41, 42], perceived barriers [45, 46], perceived benefits [42, 45, 46], cues to action [41, 42, 45], and perceived self-efficacy in dieting and exercise [42, 45] positively influence weight management intentions.

Effective weight management is an urgent requirement to prevent the serious consequences of overweight and obesity. However, to our knowledge, current weight management practices in Vietnam remain limited and have not been comprehensively studied. Although the HBM has been widely applied in international research to predict weight management behavioral intention [40–42, 45, 46], no studies have focused on applying this model in Vietnam, where sociocultural factors such as sex, marital status, educational attainment, family history, and previous weight loss experience may strongly influence behavioral intentions. Therefore, this study was conducted to identify factors influencing the weight management intentions of adults in Ho Chi Minh City through the application of the HBM and to evaluate the impact of these factors. The findings of this study will provide a crucial scientific basis for developing effective weight management intervention programs tailored to the specific cultural and social context of Vietnam, particularly in Ho Chi Minh City.

## Methods

### Study setting

A cross-sectional study was conducted among adults residing and working in Ho Chi Minh City from July to October 2023. Located in southern Vietnam, Ho Chi Minh City is the country’s leading center for economic, financial, cultural, and educational activities.

### Sample size and sampling technique

The sample size was calculated using the formula for estimating proportions in cross-sectional studies [47, 48].

$$\:n=\:\frac{{z}_{crit}^{2}\text{p}(1-\text{p})}{{e}^{2}}$$

According to this formula, a minimum sample size of 385 observations was required, based on the following assumptions: a 95% confidence interval ($\:{z}_{crit}$ = 1.96), a 50% proportion of weight management behavioral intention (*p* = 0.5, due to the lack of prior studies), and a 5% margin of error (e = 0.05). To account for potential data loss or non-responses (estimated at 10%, based on previous studies [45, 46, 49]), the final proposed sample size was determined to be 428 participants. During data collection, a sample of 428 individuals who agreed to participate was selected; however, 41 had incomplete data in the questionnaire and were excluded from the study. Therefore, the final sample size included in the analysis was 387.

Participants were selected using a quota sampling method to ensure representation of adults living and working in Ho Chi Minh City. The sample was stratified by age and educational attainment based on the results of the 2019 Vietnam Population and Housing Census [50]. Further details can be found in Supplementary Appendix 1.

### Participant selection and data collection

Participants were selected based on the following inclusion criteria: individuals aged 18 years and above, residing and working in Ho Chi Minh City, able to read and write in Vietnamese, and who provided consent to participate in the survey. The exclusion criteria included individuals who refused to participate, failed to complete the questionnaire fully, provided inaccurate information, were under 18, had a mental illness preventing them from responding, or did not reside or work in Ho Chi Minh City.

Data were collected through a self-administered online questionnaire designed to measure the components of the Health Belief Model (HBM) and the basic characteristics of the study participants (see Supplementary Appendix 2). To ensure the quality of data collection, four experienced surveyors were recruited, with responsibilities including distributing the survey and directly addressing any questions related to the survey content. These surveyors participated in a training session conducted by the principal investigator, covering topics such as the research objectives, detailed questionnaire content, procedures for managing the online survey, guidelines for obtaining informed consent, participants’ right to refuse participation, and clarifications regarding related inquiries.

### Measurement instruments

The research questionnaire was developed based on previously validated instruments used in earlier studies [40–42, 49]. The questionnaire was translated into Vietnamese and adapted to the target population and research context following international guidelines, adhering to the process of translation and cultural adaptation. This process involved the following steps: preparation, forward translation, reconciliation, back translation, review of the back translation, harmonization, pilot testing, evaluation of the pilot results, final revision, and reporting [51, 52].

The first part of the questionnaire measured the Health Belief Model (HBM) variables, including 88 statements representing eight cognitive and behavioral categories [40–42, 49], as follows: 13 questions assessed perceptions of the severity of being overweight across three subscales (emotional/mental health, physical health/fitness, and social/occupational); 7 questions evaluated perceived susceptibility to the risk of being overweight, including two subscales (lifestyle and environment); 13 questions related to perceived barriers to weight management, including three subscales (practical concerns, emotional/mental health, and cognition); 13 questions assessed perceived benefits of weight management, including three subscales (emotional/mental health, physical health/fitness, and social/occupational); 12 questions assessed cues to action, including two subscales (internal and external cues); 18 questions evaluated self-efficacy in dieting, including two subscales (habits and preferences, emotional/mental health); 7 questions assessed self-efficacy in exercise; and 5 questions measured weight management behavioral intentions, including two subscales (dieting and exercise). All statements were rated on a 5-point Likert scale, ranging from 1 (strongly disagree) to 5 (strongly agree).

The second part of the questionnaire collected basic demographic information from the participants, including age, sex, marital status, educational level, height (cm), weight (kg), and family history of obesity (particularly the obesity status of parents). This section also recorded the participants’ experience with weight loss through dieting and exercise.

### Reliability and validity

The Vietnamese version of the questionnaire was validated for content and face validity by a panel of 10 experts in the field of Health. These experts were asked to provide evaluations through suggestions and comments to refine the questionnaire. According to the methods of Lawshe and Lynn, items with a Content Validity Ratio (CVR) greater than 0.62 (for 10 experts) and a Content Validity Index (CVI) greater than 0.75 were considered acceptable [53, 54]. The CVI and CVR results indicated that all items met the required standards (CVI range: 0.8–1.00 and CVR range: 0.80–1.00). Reliability was assessed through internal consistency (Cronbach’s Alpha). The Cronbach’s alpha coefficients for the scales within the HBM (perceived threat, barriers, benefits, cues to action, and self-efficacy in dieting and exercise) ranged from 0.85 to 0.94, all exceeding the 0.7 thresholds, indicating a high level of internal reliability.

A pilot survey was conducted with a small group of 30 adults to gather feedback from respondents and make adjustments to the presentation format, wording clarity, and overall ease of understanding for the participants. To assess the stability of the questionnaire, this pilot group was asked to complete the survey again after a two-week interval. The Intraclass Correlation Coefficient (ICC) was calculated to evaluate test-retest reliability. The ICC for all items was greater than 0.75, indicating the high temporal stability of the items in the questionnaire.

### Statistical analysis

The research data were managed and analyzed using Microsoft Excel and IBM SPSS Statistics version 20. Participants’ characteristics and beliefs were described using mean values (± SD) and frequencies (percentages). Weight status was categorized into four groups: underweight (BMI < 18.5 kg/m²), normal weight (18.5 ≤ BMI < 25 kg/m²), overweight (BMI 25–29.9 kg/m²), and obese (BMI ≥ 30 kg/m²) according to the standards of the World Health Organization (WHO, 2024a, 2024b), to ensure consistency with HBM-related studies in the Asian region [42, 45, 49] and to align with the structure of the research sample. The association between participants’ basic characteristics and BMI categories was assessed using the Chi-squared test (χ2). BMI was treated as a continuous variable in the regression analysis to minimize misclassification bias.

Since the HBM variables did not follow a normal distribution, as determined by the Kolmogorov-Smirnov and Shapiro-Wilk tests, the Kruskal-Wallis test was applied to analyze the mean differences in HBM variables across weight categories. Additionally, multivariable logistic regression analysis was conducted to identify factors influencing weight management behavioral intention (the dependent variable), with independent variables including perceived threat, barriers, benefits, cues to action, self-efficacy in dieting and exercise, sex, marital status, educational attainment, family history of obesity, dieting experience (experience of diet therapy), weight loss experience through exercise, age, and BMI. The level of statistical significance was set at *p* < 0.05. The Hosmer-Lemeshow test was also used to evaluate the goodness-of-fit of the logistic regression model with the observed data.

## Results

### Characteristics of participants

A total of 387 adults, aged 18 to 70 years, completed the questionnaire, with an average age of 37.74 ± 13.56 years. The BMI of the participants, based on self-reported weight and height data, ranged from 16.2 to 34.6 kg/m², with a mean value of 22.73 ± 3.92 kg/m². Table 1 presents the basic characteristics of the participants according to the four weight status categories. The proportions of underweight, normal weight, overweight, and obese individuals were 13.2%, 60.5%, 19.1%, and 7.2%, respectively.

**Table 1:** Association between participants’ characteristics and BMI categories

Variable	All (*n* = 387)	Underweight (*n* = 51)	Normal (*n* = 234)	Overweight (*n* = 74)	Obese (*n* = 28)	*P*-value
Age Group						0.259
18 to 34	169 (43.7)	27 (16.0)	104 (61.5)	30 (17.8)	8 (4.7)	
35 to 54	153 (39.5)	13 (8.5)	93 (60.8)	32 (20.9)	15 (9.8)	
55 and older	65 (16.8)	11 (16.9)	37 (56.9)	12 (18.5)	5 (7.7)	
Sex						0.290
Male	177 (45.7)	27 (15.3)	110 (62.1)	31 (17.5)	9 (5.1)	
Female	210 (54.3)	24 (11.4)	124 (59.9)	43 (20.5)	19 (9.0)	
Marital Status						$\:{<0.001\:}^{*}$
Unmarried	206 (53.2)	32 (15.5)	139 (67.5)	23 (11.2)	12 (5.8)	
Married	181 (46.8)	19 (10.5)	95 (52.5)	51 (28.2)	16 (8.8)	
Educational Level						$\:{<0.001\:}^{*}$
Elementary School or Lower	72 (18.6)	10 (13.9)	32(44.4)	18(25.0)	12 (16.7)	
Middle School	97 (25.1)	21 (21.6)	55 (56.7)	19 (19.6)	2 (2.1)	
High School/Vocational/Technical	128 (33.1)	16 (12.5)	87 (68.0)	19 (14.8)	6 (4.7)	
College/University or Higher	90 (23.3)	4 (4.4)	60 (66.7)	18 (20.0)	8 (9.8)	
Family History of Obesity						$\:{0.005\:}^{*}$
Yes	105 (27.1)	10 (9.5)	55 (52.4)	26 (24.8)	14 (13.3)	
No	282 (72.9)	41 (14.5)	179 (63.5)	48 (17.0)	14 (5.0)	
Dieting Experience						0.061
Yes	193 (49.9)	19 (9.8)	122 (63.2)	42 (21.8)	10 (5.2)	
No	194 (50.1)	32 (16.5)	112 (57.7)	32 (16.5)	18 (9.3)	
Exercise-Based Weight Loss Experience						$\:{<0.001\:}^{*}$
Yes	209 (54.0)	11 (5.3)	131 (62.7)	51 (24.4)	16 (7.7)	
No	178 (46.0)	40 (22.5)	103 (57.9)	23 (12.9)	12 (6.7)	

*Data are presented as frequency (percentage). Significance determined by χ2 test for association with *P* < 0.05

This method of age group classification has been applied in previous studies, as referenced in the literature [55, 56]

Data analysis revealed no significant differences in weight status across age groups and sex (*p* > 0.05). This indicates that the proportions of underweight, normal weight, overweight, and obese individuals did not significantly vary by age group (*p* = 0.259) or sex (*p* = 0.290). However, marital status and educational attainment were significantly associated with weight status (*p* < 0.001). Specifically, most unmarried and married individuals had normal weight (67.5% and 52.5%). Still, the prevalence of overweight and obesity was higher among married individuals (28.2% and 8.8%) compared to unmarried individuals (11.2% and 5.8%). Additionally, the prevalence of overweight and obesity was significantly higher among those with an education level of elementary school or below (25.0% and 16.7%) compared to those with a college/university degree or higher (20.0% and 9.8%).

The association between a family history of obesity and weight status also showed significant differences (*p* = 0.005). Individuals with obese parents had a significantly higher prevalence of overweight and obesity compared to those without a family history of obesity (24.8% and 13.3% versus 17.0% and 5.0%). Although dieting experience did not significantly differ between weight status groups (*p* = 0.061), weight loss experience through exercise was markedly associated with weight status. Those with experience in weight loss through exercise had a higher prevalence of overweight and a lower prevalence of underweight compared to those without this experience (*p* < 0.001).

These results suggest that marital status, educational attainment, a family history of parental obesity, and weight loss experience through exercise are important factors significantly associated with the weight status of the study participants.

### Weight-related beliefs of participants

Table 2 details the average scores of the Health Belief Model (HBM) scales across the four weight status categories. Cronbach’s alpha coefficients range from 0.79 to 0.94, confirming the high reliability of the measurement instruments. The analysis revealed significant differences among the weight status groups regarding perceived severity, susceptibility, barriers, cues to action, self-efficacy in dieting and exercise, and weight management behavioral intentions.

**Table 2:** Comparison of HBM scale scores among BMI categories

Variable	All (*n*=387)	Underweight (*n*=51)	Normal (*n*=234)	Overweight (*n*=74)	Obese (*n=28)*	*P*-value
Perceived Severity						
Emotional/Mental Health Subscale (α = 0.83)	3.19 ± 0.95	2.64 ± 0.84	3.19 ± 1.04	3.59 ± 0.35	3.21 ± 0.93	< 0.001*
Physical Health/Fitness Subscale (α = 0.80)	3.15 ± 0.84	2.74 ± 0.73	3.10 ± 0.90	3.54 ± 0.35	3.24 ± 0.94	< 0.001*
Social/Occupational Subscale (α = 0.76)	3.18 ± 0.87	2.63 ± 0.77	3.16 ± 0.93	3.63 ± 0.40	3.14 ± 0.80	< 0.001*
Total (α = 0.92)	3.18 ± 0.81	2.67 ± 0.71	3.15 ± 0.88	3.59 ± 0.21	3.20 ± 0.81	< 0.001*
Perceived Susceptibility						
Lifestyle Subscale (α = 0.79)	3.15 ± 0.85	2.71 ± 0.79	3.13 ± 0.93	3.51 ± 0.34	3.14 ± 0.80	< 0.001*
Environmental Subscale (α = 0.68)	3.16 ± 1.00	2.60 ± 0.99	3.15 ± 1.07	3.55 ± 0.50	3.20 ± 0.91	< 0.001*
Total (α = 0.85)	3.15 ± 0.85	2.65 ± 0.82	3.13 ± 0.93	3.53 ± 0.27	3.18 ± 0.78	< 0.001*
Perceived Threat						
Total (α = 0.94)	3.16 ± 0.81	2.66 ± 0.74	3.15 ± 0.88	3.56 ± 0.19	3.19 ± 0.78	< 0.001*
Perceived Barriers						
Practical Concerns Subscale (α = 0.84)	3.50 ± 0.92	3.77 ± 0.81	3.54 ± 0.92	3.22 ± 0.94	3.39 ± 0.91	0.007*
Emotional/Mental Health Subscale (α = 0.80)	3.51 ± 0.87	3.75 ± 0.89	3.54 ± 0.89	3.30 ± 0.84	3.41 ± 0.69	0.004*
Cognitive Subscale (α = 0.89)	3.47 ± 0.87	3.84 ± 0.79	3.46 ± 0.86	3.22 ± 0.93	3.48 ± 0.82	0.001*
Total (α = 0.94)	3.49 ± 0.82	3.79 ± 0.73	3.52 ± 0.83	3.25 ± 0.83	3.43 ± 0.74	0.001*
Perceived Benefits						
Emotional/Mental Health Subscale (α = 0.83)	3.55 ± 0.81	3.26 ± 0.88	3.53 ± 0.92	3.73 ± 0.28	3.76 ± 0.44	0.134
Physical Health/Fitness Subscale (α = 0.88)	3.60 ± 0.76	3.24 ± 0.91	3.61 ± 0.85	3.79 ± 0.20	3.66 ± 0.29	0.018*
Social/Occupational Subscale (α = 0.74)	3.55 ± 0.86	3.25 ± 1.06	3.53 ± 0.94	3.80 ± 0.37	3.61 ± 0.44	0.034*
Total (α = 0.93)	3.56 ± 0.74	3.24 ± 0.90	3.56 ± 0.83	3.77 ± 0.17	3.67 ± 0.31	0.214
Cues to Action						
Internal Cues (α = 0.88)	3.37 ± 0.77	3.12 ± 0.85	3.42 ± 0.83	3.51 ± 0.29	3.10 ± 0.80	0.028*
External Cues (α = 0.87)	3.37 ± 0.75	3.09 ± 0.91	3.42 ± 0.80	3.52 ± 0.24	3.06 ± 0.74	0.013*
Total (α = 0.93)	3.37 ± 0.74	3.10 ± 0.86	3.42 ± 0.79	3.50 ± 0.23	3.08 ± 0.77	0.022*
Self-Efficacy in Dieting						
Habits and Preferences Subscale (α = 0.93)	3.43 ± 0.84	3.03 ± 0.95	3.42 ± 0.78	3.62 ± 0.85	3.69 ± 0.76	< 0.001*
Emotional/Mental Health Subscale (α = 0.81)	3.26 ± 0.86	2.83 ± 0.87	3.26 ± 0.82	3.44 ± 0.89	3.53 ± 0.87	< 0.001*
Total (α = 0.94)	3.34 ± 0.81	2.93 ± 0.86	3.34 ± 0.77	3.53 ± 0.83	3.61 ± 0.79	< 0.001*
Self-Efficacy in Exercise						
Total (α = 0.93)	3.29 ± 0.82	2.70 ± 0.74	3.35 ± 0.90	3.54 ± 0.27	3.28 ± 0.73	< 0.001*
Weight Management Behavioral Intentions						
Diet Therapy (α = 0.91)	3.08 ± 1.12	2.06 ± 0.77	3.16 ± 1.13	3.50 ± 0.83	3.23 ± 1.23	< 0.001*
Exercise Therapy (α = 0.88)	3.08 ± 1.18	1.93 ± 0.67	3.18 ± 1.20	3.52 ± 0.85	3.16 ± 1.25	< 0.001*
Total (α = 0.94)	3.08 ± 1.11	2.00 ± 0.62	3.17 ± 1.14	3.51 ± 0.75	3.19 ± 1.22	< 0.001*

α represents the Cronbach's alpha coefficient. *P*-values are based on the Kruskal-Wallis test

The perceived severity of being overweight had an average score of 3.18 ± 0.81, with significant differences among the groups (*p* < 0.001). The overweight group had the highest perception of severity (3.59 ± 0.21), while the underweight group had the lowest (2.67 ± 0.71). Similarly, perceived susceptibility to the risk of being overweight also showed significant differences (*p* < 0.001), with the overweight group feeling the most susceptible (3.53 ± 0.27) and the underweight group feeling the least at risk (2.65 ± 0.82).

Regarding perceived barriers to weight management, the average score was 3.49 ± 0.82, with the underweight group reporting the highest perceived barriers (3.78 ± 0.77). Although the perceived benefits of weight management behaviors had an average score of 3.56 ± 0.74, there were no significant differences among the groups (*p* = 0.214), except that the overweight group tended to rate social and occupational benefits higher (3.80 ± 0.37).

Cues to action had an average score of 3.37 ± 0.74, with statistically significant differences among the groups (*p* = 0.022). The normal and overweight groups responded more strongly to cues prompting weight management behaviors. Perceived self-efficacy in dieting and exercise also varied significantly among the groups, with the obese group having the highest (3.61 ± 0.79). In contrast, the underweight group had the lowest (2.93 ± 0.86).

Finally, weight management behavioral intentions, including dieting and exercise, had an average score of 3.08 ± 1.11, with significant differences among the groups (*p* < 0.001). The overweight group exhibited the strongest intentions (3.51 ± 0.75), while the underweight group had the lowest (2.00 ± 0.62), indicating a substantial discrepancy in the motivation for weight management across different weight status groups.

### Multivariable logistic regression

Multivariable logistic regression analysis explored the factors influencing weight management behavioral intentions, with detailed results in Table 3. The analysis revealed that significant factors included perceived threat, self-efficacy in exercise, sex, marital status, educational attainment, dieting experience (experience of diet therapy), and BMI, all of which significantly impacted weight management intentions (*p* < 0.05).

**Table 3:** Multivariable logistic regression analysis of variables affecting weight management behavioral intentions

Independent variable	Coefficient (β)	S.E.	*P*-value	Odds Ratio (OR)	95% C.I.	
					Lower	Upper
Perceived Threat	0.92	0.21	< 0.001*	2.51	1.65	3.81
Perceived Barriers	0.03	0.19	0.866	1.03	0.71	1.50
Perceived Benefits	0.39	0.28	0.889	1.04	0.60	1.81
Cues to Action	0.16	0.27	0.544	1.17	0.69	1.99
Self-Efficacy in Dieting	0.11	0.23	0.630	1.12	0.71	1.76
Self-Efficacy in Exercise	0.87	0.24	< 0.001*	2.39	1.48	3.84
Sex						
Male	RC	RC	RC	RC	RC	RC
Female	1.14	0.31	< 0.001*	3.13	1.70	5.77
Marital Status						
Unmarried	RC	RC	RC	RC	RC	RC
Married	−1.12	0.36	0.002*	0.33	0.16	0.65
Educational Level						
Elementary School or Lower	RC	RC	RC	RC	RC	RC
Middle School	1.32	0.50	0.008*	3.73	1.42	9.84
High School/Vocational/Technical	1.56	0.47	0.001*	4.73	1.88	11.92
College/University or Higher	1.98	0.51	< 0.001*	7.25	2.69	19.56
Family History of Obesity						
Yes	0.10	0.35	0.779	1.10	0.56	2.19
No	RC	RC	RC	RC	RC	RC
Dieting Experience						
Yes	0.61	0.31	0.049*	1.84	1.00	3.37
No	RC	RC	RC	RC	RC	RC
Exercise-Based Weight Loss Experience						
Yes	0.57	0.32	0.069	1.77	0.96	3.29
No	RC	RC	RC	RC	RC	RC
Age	−0.01	0.01	0.683	1.00	0.97	1.02
BMI	0.26	0.05	< 0.001*	1.29	1.17	1.42
Intercept	−14.44	1.96	< 0.001*			

*Abbreviation*: *RC* Reference category for categorical variables

*Significance is indicated by *P* < 0.05.

Perceived threat had a coefficient of β = 0.92 with a p-value < 0.001, indicating that for each unit increase in perceived threat, the likelihood of having an intention to manage weight increased by 2.51 times (OR = 2.51). This underscores that individuals with a higher perception of the risks associated with being overweight are more determined to manage their weight.

Perceived self-efficacy in exercise also had a significant impact, with a coefficient of β = 0.87 and a p-value < 0.001. Each unit increase in this perception increased the likelihood of having an intention to manage weight by 2.39 times (OR = 2.39). Similarly, females were 3.13 times more likely to have weight management intentions than males, with a coefficient of β = 1.14 and OR = 3.13, reflecting differences in perception and social pressures regarding weight between sexes.

Marital status emerged as another crucial factor, with married individuals less likely to have weight management intentions than unmarried individuals (β = −1.12, *p* = 0.002; OR = 0.33). Higher educational attainment was also associated with stronger weight management intentions, with coefficients ranging from β = 1.32 to 1.98 and ORs from 3.73 to 7.25, indicating that health literacy significantly promotes weight management behaviors.

Dieting experience (experience of diet therapy) and BMI were also important factors. Individuals with prior dieting experience were 1.84 times more likely to have weight management intentions (β = 0.61, *p* = 0.049). At the same time, higher BMI was associated with an increased likelihood of having such intentions, with each unit increase in BMI raising the likelihood of weight management by 1.29 times (β = 0.26, *p* < 0.001).

## Discussion

This study hypothesizes that various factors within the Health Belief Model (HBM) will significantly influence weight management behavioral intention among adults in Ho Chi Minh City. The research findings align with this hypothesis, indicating that perceptions of overweight-related threats and exercise self-efficacy drive behavioral intentions for weight management. Although numerous weight loss programs have been implemented [32, 33], achieving successful weight loss remains a significant challenge, with long-term adherence and maintenance rates often being low [34–36]. Therefore, understanding behavior and developing effective weight management strategies is paramount. The findings of this study shed light on several key factors associated with weight management intentions, thereby providing a rationale for developing more effective intervention strategies.

The results indicate that marital status and educational attainment are significantly associated with weight status. Specifically, married individuals exhibited higher rates of overweight and obesity compared to unmarried individuals, a finding consistent with previous research [42, 49, 57, 58]. This finding may reflect lifestyle and dietary habit changes following marriage, where attention to health and weight control may diminish due to family responsibilities and altered routines [57, 58]. Additionally, educational attainment was also related to weight status, with higher educational levels associated with lower rates of overweight and obesity, which aligns with previous studies [59–63]. This difference can be attributed to the fact that individuals with higher educational levels are often better equipped to access information on nutrition and health [63] and, therefore, may be more likely to engage in effective weight control practices [60].

The current findings indicate that individuals with a parent who is obese have a significantly higher prevalence of overweight and obesity compared to those without such a family history, which is consistent with previous studies [64–66]. This suggests that not only genetic predisposition [64, 65], but also food choices and eating habits shared within families [66], play an important role in shaping an individual’s risk of developing obesity. These findings highlight the need for early interventions targeting high-risk groups. Additionally, the research results indicate that individuals with prior weight loss experience through exercise exhibit higher rates of being overweight compared to those without such experience. This aligns with the findings of Doucet et al., which emphasize that while exercise supports weight management, long-term maintenance often poses challenges, leading to a higher risk of overweight recurrence [67]. However, this finding contrasts with other studies [68–70], with Blair noting that regular exercise not only supports weight loss but also helps maintain a healthy weight over time [69]. Balasekaran et al. reinforced these findings by demonstrating that weight loss due to exercise is associated with improved body composition and reduced obesity rates [68].

The study results also show that the overweight group had the highest awareness of the severity and susceptibility to the risks of being overweight. This finding aligns with Park’s research (2011), which indicated that overweight individuals often recognize the negative consequences of their weight status and express strong intentions to lose weight [41]. In contrast, underweight individuals had the lowest levels of perceived severity and susceptibility to the risk of being overweight. This result contradicts previous findings among university students [42, 45], where underweight individuals were reported to have the highest perception of overweight severity and risk. This divergence may be attributed to the greater diversity in age and educational background within our study sample. Unlike previous studies that focused on university students [42, 45], a group generally possessing higher health literacy and more frequent exposure to health-related information, our sample included participants with varied access to and understanding of such information.

Perceived barriers to weight management demonstrated significant differences across weight status groups, a finding that aligns with previous studies [42, 45, 71, 72]. This result underscores the importance of developing tailored interventions for each weight status group to optimize the effectiveness of weight management strategies. Regarding cues to action, individuals with normal weight and those who are overweight showed higher sensitivity to factors that encourage weight management behaviors than other groups. This reinforces the idea that motivational triggers substantially impact these two groups in promoting weight management behaviors. This finding is consistent with previous research [42, 73], indicating that while all weight groups are influenced by motivational factors, the degree and manner of influence differ.

Additionally, significant differences in perceived self-efficacy in dieting and exercise across weight status groups were observed, consistent with earlier studies [42, 74]. These findings suggest that self-efficacy plays a crucial role in weight management intentions, with variations in confidence levels influencing the ability to engage in and maintain healthy behaviors.

The multivariable logistic regression analysis identified several key factors significantly influencing weight management behavioral intentions, including educational attainment, sex, perceived threat, self-efficacy in exercise, dieting experience, BMI, and marital status. Notably, educational attainment emerged as the most influential factor, highlighting the critical role of education in promoting weight management intentions. This finding is consistent with previous studies, which show that individuals with higher educational levels tend to have better health awareness and more proactive weight management intentions [46, 75].

Furthermore, the perception of the threat associated with being overweight significantly influences weight management intentions, a finding consistent with previously published studies [41, 42]. Individuals with a heightened awareness of the health risks related to being overweight tend to be more determined to undertake weight management measures. Similarly, perceived self-efficacy in exercise strongly impacts weight management intentions, with those who believe in their ability to manage their weight through exercise typically being more proactive in weight management [41, 42, 45]. Sex was also identified as a significant factor, with women generally exhibiting higher weight management intentions than men, a finding that aligns with previous studies [45, 46]. This difference reflects cultural, social, and weight-related pressures, indicating that intervention strategies should be tailored to each sex to optimize the effectiveness of weight management programs.

This study’s strength lies in applying the HBM, a widely validated theoretical framework, to examine weight management behavioral intentions among adults in Ho Chi Minh City, Vietnam. By focusing on the model’s core constructs, including perceived threat, self-efficacy, perceived barriers, perceived benefits, and cues to action, the study offers a multidimensional understanding of the cognitive determinants influencing weight-related behaviors, which are often underexplored in conventional research. Moreover, it generates context-specific insights relevant to urban populations in Vietnam, where the prevalence of overweight and obesity is increasing at an alarming rate. The use of a sufficiently large sample enabled multivariable logistic regression analysis, facilitating the robust identification of key predictors such as educational attainment, perceived threat, exercise-related self-efficacy, and prior dieting experience. In addition to contributing to theoretical advancement, the study provides valuable practical implications for the development of culturally tailored intervention programs aligned with national nutrition policy priorities.

### Limitations and Future research directions

This study has several limitations that should be acknowledged. First, the cross-sectional design limits the ability to infer causal relationships between variables. Future research should adopt longitudinal approaches to track changes over time and better understand causal pathways influencing weight management behaviors. Second, the reliance on self-reported data for weight and height may introduce reporting bias. The use of objective anthropometric measurements or appropriate statistical adjustments based on demographic covariates is recommended to enhance data accuracy [76].

Additionally, the use of quota sampling and online surveys may reduce the representativeness of the sample, particularly for individuals with limited internet access or from marginalized communities. Future studies should consider employing probability-based sampling and combining both digital and non-digital data collection methods to increase inclusivity and generalizability. Moreover, while the global BMI classification from the World Health Organization (WHO) was used to maintain international comparability, it may underestimate overweight and obesity prevalence in Asian populations when compared to regional standards, such as those of the Western Pacific Regional Office (WPRO). Given the behavioral focus of this study, the WHO standard remains appropriate; however, future research should consider parallel comparisons between global and regional classification systems to improve contextual relevance.

Addressing these limitations in future studies will strengthen methodological rigor, improve external validity, and support the development of culturally appropriate, evidence-based interventions for weight management in Vietnam and similar settings.

### Practical implications of the study

These findings align with Vietnam’s National Nutrition Strategy for 2021–2030 and Vision for 2045 [77], particularly in addressing overweight and obesity issues through educational and behavioral change programs. This study provides a scientific basis to support and enhance existing policies and intervention programs, emphasizing the importance of personalizing weight management programs. Specifically, the results indicate the necessity of designing educational initiatives to enhance health literacy, increase awareness of the risks of being overweight, and improve self-efficacy in exercise. Furthermore, tailoring interventions based on individual characteristics such as sex, marital status, weight loss experience through dieting, and BMI will optimize effectiveness, ensuring the program meets the needs and capabilities of different target groups, thereby enhancing weight management efficacy among adults in Ho Chi Minh City.

## Conclusion

This study has identified key factors influencing weight management behavioral intentions among adults in Ho Chi Minh City by applying the Health Belief Model (HBM). Among the factors analyzed, educational attainment emerged as the most influential, highlighting the crucial role of education in enhancing awareness and promoting effective weight management strategies. In addition, perceived threat and self-efficacy in exercise also play significant roles in shaping weight management intentions, though their influence is not as strong as that of educational attainment. Other factors, such as sex, marital status, and BMI, also showed statistically significant associations with weight behavioral management intentions. However, as these are non-modifiable characteristics, they are not intended as targets for behavioral intervention. Instead, they offer valuable insight into subgroup differences, supporting the development of tailored strategies that are more culturally and socially responsive.

The study’s results not only contribute to a deeper understanding of weight management behaviors but also provide a crucial scientific basis for developing culturally and socially relevant intervention programs in Vietnam, particularly in urban areas like Ho Chi Minh City. This research holds significant implications for clinicians and policymakers, aiding them in offering more effective counseling to patients and guiding public health education policies. Additionally, the study underscores the necessity for systemic solutions that address the root causes of overweight and obesity rather than solely focusing on individual efforts, thereby encouraging policies that promote broader, more sustainable action.

## Supplementary Information

Supplementary Material 1.

Supplementary Material 2.

## Data Availability

Data are available from the authors upon reasonable request. Contact: Thang T. Vo, email: thangvt@ueh.edu.vn, address: 279 Nguyen Tri Phuong, Room 10.04, District 10, Ho Chi Minh City, Vietnam.
