# Supplementary material for: Application of the health belief model to study weight management behavioral intentions among adults in Ho Chi Minh City, Vietnam: a cross-sectional study
Source: BMC Public Health. 2025 Jul 11;25:2436. doi: 10.1186/s12889-025-23637-9 (PMC12247397; doi:10.1186/s12889-025-23637-9)
Supplement: Supplementary file 1 — Supplementary Material 1. [file 12889_2025_23637_MOESM1_ESM.docx]

**QUESTIONNAIRE**

**Dear Sir/Madam,**

I am __________________, a researcher from the University of Economics Ho Chi Minh City (UEH). I seek your participation in this study and would greatly appreciate your interest and involvement. Please allow me to explain the research details and how you can participate.

**Study Title**: Application of the Health Belief Model to Study Weight Management Behavioral Intentions Among Adults in Vietnam: A Cross-Sectional Study.

**The study's objective**: This study aims to identify the factors influencing adults' weight management behavioural intentions in Ho Chi Minh City through the Health Belief Model (HBM) and evaluate their impact.

**Procedure and time**: This study involves completing a self-administered questionnaire about the Health Belief Model (HBM) and your basic information, which will take approximately 15-30 minutes.

**Confidentiality**: All provided information will be kept confidential, ensuring anonymity and non-disclosure of personal identifiers. The research results will focus solely on the study’s objectives, and no written or oral reports will be able to link participants with the collected data.

**Rights**: Participation is entirely voluntary. You may choose to participate or not without affecting any of your community benefits. If you decide to participate, you may withdraw at any time without explanation and without losing any benefits. You may also ask questions for clarification and refuse to answer any questions that make you uncomfortable.

If you have any questions or concerns about the study, please get in touch with Associate Professor Dr Thang Vo at +84934051018.

Have you read the above information and agreed to participate in this study?

- Agree
- Disagree

Where are you currently living and working?

- Ho Chi Minh City
- Outside of Ho Chi Minh City

**PART 1: HEALTH BELIEF MODEL**

| - 1. **Perceived Severity of Overweight**   1 = Strongly Disagree; 2 = Disagree; 3 = Neutral; 4 = Agree; 5 = Strongly Agree | | | | | | |
| --- | --- | --- | --- | --- | --- | --- |
| **No.** | **Question** | **Answer (Please choose the appropriate column)** | | | | |
| **Emotional/Mental Health Subscale** | | | | | | |
| 1 | Being overweight can make me feel anxious and stressed. | 1 | 2 | 3 | 4 | 5 |
| 2 | Being overweight can make others perceive me as less physically attractive. | 1 | 2 | 3 | 4 | 5 |
| 3 | Being overweight can make me feel unhappy and depressed. | 1 | 2 | 3 | 4 | 5 |
| 4 | Being overweight can reduce my self-confidence. | 1 | 2 | 3 | 4 | 5 |
| **Physical Health Subscale** | | | | | | |
| 5 | Being overweight can make it harder to engage in physical activities or sports that I enjoy. | 1 | 2 | 3 | 4 | 5 |
| 6 | Being overweight can make it more difficult for me to get enough sleep. | 1 | 2 | 3 | 4 | 5 |
| 7 | Being overweight can cause knee pain. | 1 | 2 | 3 | 4 | 5 |
| 8 | Being overweight can negatively affect my health in the future. | 1 | 2 | 3 | 4 | 5 |
| 9 | Being overweight can increase the risk of diabetes, high blood pressure, cancer, and other diseases. | 1 | 2 | 3 | 4 | 5 |
| **Social/Occupational Subscale** | | | | | | |
| 10 | Being overweight can reduce my chances of success in social relationships. | 1 | 2 | 3 | 4 | 5 |
| 11 | Being overweight can affect my job performance and career advancement. | 1 | 2 | 3 | 4 | 5 |
| 12 | Being overweight can reduce satisfaction and enjoyment in social activities. | 1 | 2 | 3 | 4 | 5 |
| 13 | Being overweight can prevent me from wearing the clothes I want. | 1 | 2 | 3 | 4 | 5 |

| - 1. **Perceived Susceptibility to Overweight**   1 = Strongly Disagree; 2 = Disagree; 3 = Neutral; 4 = Agree; 5 = Strongly Agree | | | | | | |
| --- | --- | --- | --- | --- | --- | --- |
| **No.** | **Question** | **Answer (Please choose the appropriate column)** | | | | |
| **Lifestyle Subscale** | | | | | | |
| 1 | I am more likely to become overweight if I engage in less than 30 minutes of moderate-intensity physical activity daily. | 1 | 2 | 3 | 4 | 5 |
| 2 | I am more likely to become overweight if I consume sugary drinks, food, or snacks daily. | 1 | 2 | 3 | 4 | 5 |
| 3 | I am more likely to become overweight if I eat fried foods or snacks daily. | 1 | 2 | 3 | 4 | 5 |
| 4 | I am more likely to become overweight if I eat fast food three or more times per week. | 1 | 2 | 3 | 4 | 5 |
| 5 | I am more likely to become overweight if I do not pay attention to how much I eat or drink. | 1 | 2 | 3 | 4 | 5 |
| **Environmental Subscale** | | | | | | |
| 6 | I am more likely to become overweight if one or both of my parents are overweight or obese. | 1 | 2 | 3 | 4 | 5 |
| 7 | I am more likely to become overweight if I have a genetic predisposition to being overweight or obese. | 1 | 2 | 3 | 4 | 5 |

| - 1. **Perceived Barriers to Weight Management**   1 = Strongly Disagree; 2 = Disagree; 3 = Neutral; 4 = Agree; 5 = Strongly Agree | | | | | | |
| --- | --- | --- | --- | --- | --- | --- |
| **No.** | **Question** | **Answer (Please choose the appropriate column)** | | | | |
| **Practical Concerns Subscale** | | | | | | |
| 1 | Low-calorie drinks, foods, and snacks are too expensive for my budget. | 1 | 2 | 3 | 4 | 5 |
| 2 | Grocery shopping and preparing healthy meals will take too much of my time. | 1 | 2 | 3 | 4 | 5 |
| 3 | Daily exercise/physical activity will reduce the time I have for work and family. | 1 | 2 | 3 | 4 | 5 |
| 4 | My work/study is more important than maintaining a healthy diet and physical activity. | 1 | 2 | 3 | 4 | 5 |
| **Emotional/Mental Health Subscale** | | | | | | |
| 5 | I have no motivation to adopt healthy eating and physical activity habits. | 1 | 2 | 3 | 4 | 5 |
| 6 | I prefer eating fried foods and snacks over grilled or steamed dishes. | 1 | 2 | 3 | 4 | 5 |
| 7 | I prefer consuming sugary drinks, food, and snacks over low-calorie options. | 1 | 2 | 3 | 4 | 5 |
| 8 | I often turn to food when I feel stressed or need comfort. | 1 | 2 | 3 | 4 | 5 |
| **Knowledge Subscale** | | | | | | |
| 9 | I do not know where to find accurate information on achieving and maintaining a healthy weight. | 1 | 2 | 3 | 4 | 5 |
| 10 | I do not know how to fit physical activity into my daily schedule. | 1 | 2 | 3 | 4 | 5 |
| 11 | I do not know where to buy healthy drinks and foods. | 1 | 2 | 3 | 4 | 5 |
| 12 | I do not know how to prepare low-calorie drinks, foods, or snacks. | 1 | 2 | 3 | 4 | 5 |
| 13 | I do not know how to choose low-calorie drinks, foods, or snacks. | 1 | 2 | 3 | 4 | 5 |

| - 1. **Perceived Benefits of Weight Management**   1 = Strongly Disagree; 2 = Disagree; 3 = Neutral; 4 = Agree; 5 = Strongly Agree | | | | | | |
| --- | --- | --- | --- | --- | --- | --- |
| **No.** | **Question** | **Answer (Please choose the appropriate column)** | | | | |
| **Emotional/Mental Health Subscale** | | | | | | |
| 1 | Adopting healthy eating and physical activity habits helps me reduce stress and anxiety. | 1 | 2 | 3 | 4 | 5 |
| 2 | Adopting healthy eating and physical activity habits improves my body image. | 1 | 2 | 3 | 4 | 5 |
| 3 | Adopting healthy eating and physical activity habits enhances my self-esteem. | 1 | 2 | 3 | 4 | 5 |
| 4 | Adopting healthy eating and physical activity habits improves my mood. | 1 | 2 | 3 | 4 | 5 |
| **Physical Health Subscale** | | | | | | |
| 5 | Adopting healthy eating and physical activity habits makes it easier for me to engage in physical exercise or sports I enjoy. | 1 | 2 | 3 | 4 | 5 |
| 6 | Adopting healthy eating and physical activity habits makes me feel more energetic. | 1 | 2 | 3 | 4 | 5 |
| 7 | Adopting healthy eating and physical activity habits increases my chances of having good health now and in the future. | 1 | 2 | 3 | 4 | 5 |
| 8 | Adopting healthy eating and physical activity habits helps improve a health condition or issue I currently have. | 1 | 2 | 3 | 4 | 5 |
| 9 | Adopting healthy eating and physical activity habits improves my ability to work more effectively. | 1 | 2 | 3 | 4 | 5 |
| 10 | Adopting healthy eating and physical activity habits helps me sleep better. | 1 | 2 | 3 | 4 | 5 |
| 11 | Adopting healthy eating and physical activity habits helps me complete daily activities more easily. | 1 | 2 | 3 | 4 | 5 |
| **Social/Occupational Subscale** | | | | | | |
| 12 | I will change my eating and physical activity habits if I am dissatisfied with my appearance when looking in the mirror. | 1 | 2 | 3 | 4 | 5 |
| 13 | Adopting healthy eating and physical activity habits makes me feel more comfortable when around others. | 1 | 2 | 3 | 4 | 5 |

| - 1. **Cues to Action**   1 = Strongly Disagree; 2 = Disagree; 3 = Neutral; 4 = Agree; 5 = Strongly Agree | | | | | | |
| --- | --- | --- | --- | --- | --- | --- |
| **No.** | **Question** | **Answer (Please choose the appropriate column)** | | | | |
| **Internal Cues Subscale** | | | | | | |
| 1 | I will adopt healthy eating and physical activity habits if I look in the mirror and feel dissatisfied with my body. | 1 | 2 | 3 | 4 | 5 |
| 2 | I will adopt healthy eating and physical activity habits if my clothes feel uncomfortably tight. | 1 | 2 | 3 | 4 | 5 |
| 3 | I will adopt healthy eating and physical activity habits if I experience a health issue that can be improved by a healthy weight. | 1 | 2 | 3 | 4 | 5 |
| 4 | I will change my eating and physical activity habits if I feel judged unfairly because of my weight. | 1 | 2 | 3 | 4 | 5 |
| 5 | I will adopt healthy eating and physical activity habits if achieving a healthy weight helps me meet my personal or professional goals. | 1 | 2 | 3 | 4 | 5 |
| 6 | I will change my eating and physical activity habits if I know that a healthy weight can improve my depression, anxiety, or stress. | 1 | 2 | 3 | 4 | 5 |
| **External Cues Subscale** | | | | | | |
| 7 | I will adopt healthy eating and physical activity habits if a doctor/nurse/dietitian advises me to achieve a healthy weight. | 1 | 2 | 3 | 4 | 5 |
| 8 | I will adopt healthy eating and physical activity habits if a relative has a serious illness due to being overweight or obese. | 1 | 2 | 3 | 4 | 5 |
| 9 | I will adopt healthy eating and physical activity habits if a family member or close friend advises me to maintain a healthy weight. | 1 | 2 | 3 | 4 | 5 |
| 10 | I will change my eating and physical activity habits if I take a class providing information about the health risks of being overweight or obese. | 1 | 2 | 3 | 4 | 5 |
| 11 | I will change my eating and physical activity habits if I hear information on the radio, television, or social media about the health risks of being overweight or obese. | 1 | 2 | 3 | 4 | 5 |
| 12 | I will adopt healthy eating and physical activity habits if I see an advertisement for a product or service that claims to help me achieve a healthy weight. | 1 | 2 | 3 | 4 | 5 |

| - 1. **Self-Efficacy for Dieting**   1 = Strongly Disagree; 2 = Disagree; 3 = Neutral; 4 = Agree; 5 = Strongly Agree | | | | | | |
| --- | --- | --- | --- | --- | --- | --- |
| **No.** | **Question** | **Answer (Please choose the appropriate column)** | | | | |
| **Habits and Preferences Subscale** | | | | | | |
| 1 | I can maintain eating three regular meals every day. | 1 | 2 | 3 | 4 | 5 |
| 2 | I can control the portion size of each meal. | 1 | 2 | 3 | 4 | 5 |
| 3 | I can choose to eat fresh foods rather than processed ones. | 1 | 2 | 3 | 4 | 5 |
| 4 | I can limit eating sweets such as candies and cakes. | 1 | 2 | 3 | 4 | 5 |
| 5 | I can limit eating fried and greasy foods. | 1 | 2 | 3 | 4 | 5 |
| 6 | I can limit drinking carbonated beverages such as sodas. | 1 | 2 | 3 | 4 | 5 |
| 7 | I can eat a variety of foods to maintain a balanced diet. | 1 | 2 | 3 | 4 | 5 |
| 8 | I can avoid eating right before going to bed. | 1 | 2 | 3 | 4 | 5 |
| 9 | I can eat slowly even when I am hungry. | 1 | 2 | 3 | 4 | 5 |
| 10 | I can stop eating before I am full, even if the food is very delicious. | 1 | 2 | 3 | 4 | 5 |
| 11 | I can wake up early to have breakfast every day. | 1 | 2 | 3 | 4 | 5 |
| 12 | I can focus on eating without watching TV or reading. | 1 | 2 | 3 | 4 | 5 |
| 13 | I can refuse food when I am not hungry, even if others offer it to me. | 1 | 2 | 3 | 4 | 5 |
| **Emotional/Mental Health Subscale** | | | | | | |
| 14 | I can avoid eating when I feel bored. | 1 | 2 | 3 | 4 | 5 |
| 15 | I can avoid eating when I am only slightly hungry. | 1 | 2 | 3 | 4 | 5 |
| 16 | I can avoid eating when I am angry. | 1 | 2 | 3 | 4 | 5 |
| 17 | I can avoid eating when I am depressed. | 1 | 2 | 3 | 4 | 5 |
| 18 | I can avoid eating when I am anxious or excited. | 1 | 2 | 3 | 4 | 5 |

| - 1. **Self-Efficacy for Exercise**   1 = Strongly Disagree; 2 = Disagree; 3 = Neutral; 4 = Agree; 5 = Strongly Agree | | | | | | |
| --- | --- | --- | --- | --- | --- | --- |
| **No.** | **Question** | **Answer (Please choose the appropriate column)** | | | | |
| 1 | I can walk a long distance in about 15 minutes. | 1 | 2 | 3 | 4 | 5 |
| 2 | I can exercise until I am out of breath and still maintain the activity. | 1 | 2 | 3 | 4 | 5 |
| 3 | I can exercise in cold weather. | 1 | 2 | 3 | 4 | 5 |
| 4 | I can exercise in hot weather. | 1 | 2 | 3 | 4 | 5 |
| 5 | I can exercise with friends. | 1 | 2 | 3 | 4 | 5 |
| 6 | I can choose to exercise instead of watching TV when I have free time. | 1 | 2 | 3 | 4 | 5 |
| 7 | I can take the stairs instead of the elevator when possible. | 1 | 2 | 3 | 4 | 5 |

| - 1. **Behavioral Intentions for Weight Management**   1 = Strongly Disagree; 2 = Disagree; 3 = Neutral; 4 = Agree; 5 = Strongly Agree | | | | | | |
| --- | --- | --- | --- | --- | --- | --- |
| **No.** | **Question** | **Answer (Please choose the appropriate column)** | | | | |
| **Dieting Subscale** | | | | | | |
| 1 | I intend to control my diet to lose weight within the next six months. | 1 | 2 | 3 | 4 | 5 |
| 2 | I intend to consult a nutritionist to lose weight within the next six months. | 1 | 2 | 3 | 4 | 5 |
| 3 | I intend to join an obesity-related class if one is available in my area within the next six months. | 1 | 2 | 3 | 4 | 5 |
| **Exercise Subscale** | | | | | | |
| 4 | I intend to regularly exercise by myself to lose weight within the next six months. | 1 | 2 | 3 | 4 | 5 |
| 5 | I intend to join a fitness center or a class to lose weight within the next six months. | 1 | 2 | 3 | 4 | 5 |

| **PART 2: PARTICIPANT CHARACTERISTICS** | | |
| --- | --- | --- |
| **No.** | **Question** | **Answer** |
| 1 | What is your year of birth? | ………………. |
| 2 | What is your gender? | - Male - Female |
| 3 | What is your marital status? | - Single - Married |
| 4 | What is your highest level of education? | Primary school graduate or lower  Secondary school graduate   - High school graduate/Vocational training - College/University graduate or higher |
| 5 | What is your height (in cm)? | ………………. |
| 6 | What is your weight (in kg)? | ………………. |
| 7 | Does any member of your family (father/mother) have obesity? | - No - Yes |
| 8 | Do you have experience losing weight through dieting? | - No - Yes |
| 9 | Do you have experience losing weight through exercise? | - No - Yes |
