# Supplementary material for: Application of the health belief model to study weight management behavioral intentions among adults in Ho Chi Minh City, Vietnam: a cross-sectional study
Source: BMC Public Health. 2025 Jul 11;25:2436. doi: 10.1186/s12889-025-23637-9 (PMC12247397; doi:10.1186/s12889-025-23637-9)
Supplement: Supplementary file 2 — Supplementary Material 2. [file 12889_2025_23637_MOESM2_ESM.docx]

**Appendix 1.**

Table A1: Participant recruitment using a stratified quota sampling technique based on age and education, according to the 2019 Vietnam Population and Housing Census results.

| **Age** **Education** | **Population** | | | **Proportion** | | | **Sample** | | | |
| --- | --- | --- | --- | --- | --- | --- | --- | --- | --- | --- |
|  | **18-34** | **35-54** | **55 +** | **18-34** | **35-54** | **55 +** | **18-34** | **35-54** | **55 +** | **Total** |
| Primary school graduate or lower | 281,153 | 576,232 | 485,237 | 0.04 | 0.08 | 0.07 | 17 | 36 | 30 | 83 |
| Secondary school graduate | 686,934 | 809,935 | 320,259 | 0.10 | 0.12 | 0.05 | 43 | 50 | 20 | 113 |
| High school graduate/Vocational training | 1,162,999 | 761,064 | 320,280 | 0.17 | 0.11 | 0.05 | 72 | 47 | 20 | 139 |
| College/University graduate or higher | 841,606 | 509,727 | 148,117 | 0.12 | 0.07 | 0.02 | 52 | 32 | 9 | 93 |
| Total | 2,972,693 | 2,656,958 | 1,273,892 | 0.43 | 0.38 | 0.18 | 184 | 165 | 79 | 428 |

To ensure the sample adheres to the quota sampling method when using online surveys:

1. Quota Design: The total sample size (428) is divided into 12 quotas based on age groups (18-34, 35-54, 55+) and educational levels (elementary school or lower, secondary school, high school/vocational school, college/university).

Table A2. Twelve sample quotas based on age groups and educational levels

| Age Group Educational Level | **Sample** | | | |
| --- | --- | --- | --- | --- |
|  | **18-34** | **35-54** | **55 +** | **Tổng** |
| Elementary School or Lower | 17 | 36 | 30 | 83 |
| Middle School | 43 | 50 | 20 | 113 |
| High School/  Vocational/Technical | 72 | 47 | 20 | 139 |
| College/University  or Higher | 52 | 32 | 9 | 93 |
| Total | 184 | 165 | 79 | 428 |

1. Sample allocation for surveyors: Four surveyors, each in charge of an area, collected 107 responses, adhering to specific quotas (e.g., 4-5 responses from the "18-34 x elementary or lower" group, 9 responses from the "35-54 x elementary or lower" group, 7-8 responses from the "55+ x elementary or lower" group, etc.).
2. Real-time quota monitoring: The online system tracked the number of responses. When a quota was reached, the system notified the surveyor and locked the quota to prevent over-collection.
3. Quality control: Investigators monitored data to ensure compliance and accuracy. Automatic checks in the online system helped maintain quota adherence.
